# Dietary energy density, metabolic parameters, and blood pressure in a sample of adults with obesity

**DOI:** 10.1186/s12902-022-01243-9

**Published:** 2023-01-05

**Authors:** Mohammad-Sadegh Pour-Abbasi, Negin Nikrad, Mahdieh Abbasalizad Farhangi, Sahar Vahdat, Faria Jafarzadeh

**Affiliations:** 1 Department of CardioVascular Surgery, Kashan University of Medical Sciences, Kashan, Iran; 2 Department of Community Nutrition, Faculty of Nutrition, Tabriz University of Medical Sciences, Tabriz, Iran; 3 Tabriz Health Services Management Research Center, Tabriz University of Medical Sciences, Attar Neyshabouri St, Tabriz, Iran; 4 Isfahan Kidney Disease Research Center, Khorshid Hospital, School of Medicine, Isfahan University of Medical Sciences, Isfahan, Iran; 5 Department of Internal Medicine, School of Medicine, North Khorasan University of Medical Sciences, Bojnourd, Iran

**Keywords:** Dietary energy density, Metabolic syndrome, Blood pressure, Obesity, Cardio-metabolic risk factors

## Abstract

**Background:**

Several previous studies revealed the role of dietary energy density (DED) in developing obesity and related disorders. However, the possible role of DED in triggering cardiometabolic risk factors of individuals with obesity has not been studied yet. This study aimed to evaluate the association between DED and anthropometric parameters, blood pressure, and components of metabolic syndrome (MetS) (such as glycemic markers, lipid profile, and blood pressure) among individuals with obesity.

**Methods:**

In this cross-sectional study, we included 335 adults with obesity (BMI ≥ 30 kg/m^2^) aged 20–50 years in Tabriz and Tehran, Iran. Dietary intake was assessed by a validated semi-quantitative Food Frequency Questionnaire (FFQ), including 168 food items; then, DED was calculated. MetS was defined based on the guidelines of the National Cholesterol Education Program Adult Treatment Panel III (NCEP-ATP III). Enzymatic methods were used to assess serum lipids, glucose, and insulin concentrations. Blood pressure was measured by sphygmomanometer and body composition by bioelectrical impedance analysis (BIA).

**Results:**

Participants in the higher tertiles of DED had more intake of carbohydrate, dietary fat, saturated fatty acid (SFA), monounsaturated fatty acid (MUFA), polyunsaturated fatty acid (PUFA), and meat, fish, poultry (MFP). Increasing the DED in both methods had no association with systolic blood pressure (SBP), fasting blood sugar (FBS), low-density lipoprotein cholesterol (LDL-C), insulin, triglyceride (TG), and homeostatic model assessment of insulin resistance (HOMA-IR) even after adjustment for confounders. However, diastolic blood pressure (DBP) decreased in second tertile of DED I (β = 0.921, *P* = 0.004). The quantitative insulin-sensitivity check index (QUICKI) in second tertile of both DED methods had significant positive association with DED. In the second tertile of DED II, while total cholesterol (TC) significantly decreased (P crude = 0.036, P adjusted = 0.024), high-density lipoprotein cholesterol (HDL-C) increased (β = 1.096, *p* = 0.03). There was no significant changes in biochemical parameters in third tertile of DED I and II even after adjustment for covariates. Also, higher tertiles of DED was associated with reduced prevalence of MetS.

**Conclusion:**

High DED was associated with lower levels of blood pressure and TC but elevated levels of HDL and QUICKI independent of such confounders as age, BMI, sex, and physical activity. Further longitudinal studies are warranted to better elucidate casual associations.

## Background

Obesity is associated with numerous health problems and individuals with obesity are at greater risk of type two diabetes (T2D), metabolic syndrome (MetS), cardiovascular events, and cancers [1–3]. The prevalence of obesity is raising worldwide, attracting public concern. In 2014, there were more than 1.9 billion overweight and 600 million adults with obesity worldwide [4]. This growing prevalence is mostly due to changes in lifestyle and dietary intakes [5]. Similarly, in Iran, the combined prevalence of overweight and obesity is as high as 76% in some regions [6]. Obesity is a major risk factor for MetS, which is associated with glucose intolerance and insulin resistance, central obesity, disturbed serum lipids, and high blood pressure [7].

Diet is a modifiable risk factor of chronic diseases. Numerous recent studies focused on the role of healthy adequate diet in diet-disease relationships [8, 9]. Most of the studies evaluated the relationship between isolate dietary ingredients (e.g., isolate effects of vitamins or minerals) [10–12] or the role of dietary patterns [13, 14] and indices (e.g., glycemic indices, inflammatory indices, etc.) [15, 16] in developing obesity and metabolic disorders.

Recently, the dietary energy density (DED) has received considerable attention in relation to obesity and MetS. DED is defined as the amount of energy per unit weight of a food or beverage, and is usually reported as kilocalories/100 g of food [17]. While some studies excluded beverages in DED calculation [18, 19], some others considered all the food items [20, 21]. Although DED is defined as the energy density of a total diet, there is no consensus about the appropriate method of ED calculation. The studies that excluded beverages from DED calculation mentioned that beverages intake is highly variable and DED calculation based on inclusion of beverages might diminish its association with health outcomes [22]. However, beverages are an important part of usual dietary intakes and excluding them is not logical scientifically. For example, people living in Tabriz, Iran have a high tendency to drink sweet beverages [23, 24].

High-energy-dense diets are rich in energy and fat, but low in fruits, vegetables and fiber. Moreover, higher DED is negatively associated with diet quality, which promotes weight gain [25]. It has been reported that higher DED is associated with the risk of obesity [26, 27] and obesity-related disorders [28], indicating that adherence to diets with lower DED might be an important preventive strategy for obesity-related comorbidities [29]. A systematic review and meta-analysis study revealed that a high-energy-dense diet was directly associated with weight gain and risk of elevated adiposity [25]. Higher content of refined grains and added sugars of high-energy-dense diets may contribute to the development of insulin resistance, which is a basic abnormality of the MetS. Furthermore, high consumption of saturated and trans-fatty acids in energy-dense diets might explain their effects on insulin resistance and MetS [26].

Several studies with inconsistent results evaluated the association between DED and health outcomes. While one study reported the effects of diets with high DED in increasing adiposity and weight gain among patients with diabetes [22], several other studies did not show any association between DED and obesity or BMI in general population [18, 29, 30]. Similarly, for the association between DED and metabolic disorders, several studies revealed the positive association between diets with high DED and odds of MetS in adults [26, 31]. Furthermore, several other studies revealed the negative association between DED and blood pressure in general populations [22, 30]. Takeda et al*.* reported that increased DED was associated with increased HbA1c and non-HDL cholesterol among patients with T2D [22]. Dietary patterns are most likely to vary by gender, socioeconomic status, and ethnicity [32]. Despite the fact that Iran is a multi-ethnic country, most previous studies of Iranian dietary patterns were conducted with residents of the capital city, Tehran, without taking ethnicity into account [26, 30, 33, 34]. Moreover, differences in BMI between ethnic groups can be explained in part by the overall DED of their traditional diets [35]. Due to the limited number of studies in this regard, it is difficult to infer a scientific conclusion.

In the literature review phase, we did not find any study evaluating the relationship between DED and all components of MetS among adults with obesity in Tabriz and Tehran, Iran. Therefore, we aimed to evaluate the association between DED and components of MetS (such as glycemic markers, lipid profile, and blood pressure) among apparently healthy Iranian adults with obesity. Meanwhile, we assessed the association between tertiles of DED and dietary intake among this population.

## Methods and materials

### Participants

In this cross-sectional study, we included a total of 335 individuals with obesity in Tabriz and Tehran, Iran who were participated in two previous projects [36, 37]. The sample size was calculated by using single proportion formula $n=\frac{({\mathrm{Z\alpha }/2)}^{2}\mathrm{p}(1-\mathrm{p})}{{d}^{2}}$ [38], according to the prevalence of MetS (27%) in Iranian adults [39], an error coefficient of d = 0**•**05 and at α level of 0•05. Accordingly, the calculated sample size was estimated as 302 individuals. Considering the drop-out rate of 11%, the final sample size of 335 participants was estimated. The participants were recruited from the combination of two projects among individuals with obesity. The subjects were invited by public announcements and were included if they met the inclusion criteria (age range: 20–50 years old, BMI ≥ 30 kg/m^2^). The exclusion criteria were being pregnant, lactating, menopausal, having recent bariatric surgery, cardiovascular diseases, cancer, hepatic and renal diseases, diabetes mellitus, and taking any medications affecting weight.

An informed written consent was obtained from all participants and the study proposal was approved by the Ethics Committee of Tabriz University of Medical Sciences, Tabriz, Iran (code: IR.TBZMED.REC.1401.648).

### General characteristics and anthropometric assessments

Using a questionnaire, we collected sociodemographic information, including sex, age, smoking status, educational level, marital status, occupation, medical history, and family size, and calculated the socioeconomic status (SES) score. SES was determined through considering such factors as educational level, occupational position, house ownership, and family size. Education was measured as a categorical variable (highest level of educational attainment). This variable was recorded based on a 5-point Likert scale ranging from 0 to 5 (illiterate: 0; less than diploma: 1; diploma and associate degree: 2; bachelor’s degree: 3; master’s degree: 4; and higher: 5). The occupation of female subjects was categorized into five groups, including housewife, employee, student, self-employed, and others. The occupation of male subjects was categorized as follows: unemployed: 1, worker, farmer, and rancher: 2, others: 3, employee: 4, and self-employed: 5. Accordingly, participants were categorized as ≤ 3, 4–5, ≥ 6 in terms of family size. Besides, they were given scores 1 and 2 if they were tenant and landlord, respectively. Next, each participant received a score between 1 and 15 for the whole SES score. A short version of the International Physical Activity Questionnaire (IPAQ) was used to assess the physical activity level of participants [40]. Using visual analogue scale (VAS), the state of the appetite was assessed in fasting state in the morning. The VAS was calculated by marking a 100-mm line at each end of the line with the opposing words "I'm not at all hungry" and "I have not been so hungry." This questionnaire asked about cravings for sweet, salty, and fatty foods as well as hunger, satiety, fullness, and future food intake [41]. The distance between the left side of the line and the mark was used to determine appetite. Body composition was measured by bioelectrical impedance analysis (BIA) method (Tanita, BC-418 MA, Tokyo, Japan). This device calculates body fat percentage, fat mass (FM), fat free mass (FFM), and predicted muscle mass. The participants’ height and weight were measured using a wall-mounted stadiometer and a Seca scale (Seca co., Hamburg, Germany) to the nearest 0.5 cm and 0.1 kg, respectively. The short form of the IPAQ was used for physical activity assessment [42]. Waist circumference (WC) was measured at the midpoint between the lower costal margin and the iliac crest using a tape measure to the nearest 0.1 cm while hip circumference (HC) was measured over the widest part of the buttocks and was recorded to the nearest 0.1 cm. Body mass index (BMI) and waist-to-hip ratio (WHR) were calculated. Blood pressure was measured with a standard mercury sphygmomanometer twice in the same arm after at least 15 min of rest; the mean of the two measurements was used for analysis. MetS was defined according to the NCEP-ATP III criteria [43].

### Dietary assessments

Dietary information was collected using a validated semi-quantitative Food Frequency Questionnaire (FFQ), adapted for Iranian population [44]. Mirmiran et al*.* assessed the reliability and relative validity of the FFQ developed for the Tehran Lipid and Glucose Study (TLGS). The agreement percentages ranged from 39.6 to 68.3% in men and from 39.6 to 54.1% in women. The mean adjusted intraclass correlation coefficients between the two FFQs was 0.48. So, the FFQ used in the TLGS had a reasonable relative validity and reliability for nutrient intakes in Iranian adults [45]. Participants were asked to report frequency and amount of each food item consumed on a daily, weekly, monthly, or yearly basis. Then, the reported frequency of consumed foods and portion sizes for each food item were converted to gram using household measures. For calculation of DED, the daily energy intake of each individual food item (kcal/day) was divided by the total weight of consumed foods (g/d) [30].

### Calculation of DED

The ratio of energy [kcal] to weight [g] is known as the dietary energy density, which is constant regardless of amounts consumed. In the current study, daily energy density value was calculated by dividing total daily energy intake by the weight of all the food and beverages consumed. Two methods were used to obtain ED: DED I, which considers dietary energy density from foods and all beverages (carbonated drinks, fruit juice and fruit-flavored drinks, milk, tea, and coffee) and DED II, which considers dietary energy density only from foods and not beverages [46].

### Biochemical assessment

For sampling, 10 ml of venous blood was obtained from all subjects and centrifuged at 4500 rpm for 10 min to separate serum and plasma samples. Serum total cholesterol (TC), triglyceride (TG), high-density lipoprotein cholesterol (HDL-C), and fasting blood sugar (FBS) were evaluated using a commercial kit (Pars Azmoon, Tehran, Iran). Furthermore, low-density lipoprotein cholesterol (LDL-C) level was estimated by the Sampson Eq. [47]. Enzyme-linked immunosorbent assay (ELISA) kits were used to measure serum insulin, concentrations (Bioassay Technology Laboratory, Shanghai Korean Biotech, Shanghai City, China). Homeostatic model assessment for insulin resistance (HOMA-IR) was calculated using the formula: fasting insulin (μ IU/ml) × fasting glucose (mmol/l) /22.5 and quantitative insulin sensitivity check index (QUICKI) as 1/ [log fasting insulin (μU/mL) + log glucose (mmol/L)].

### Statistical analyses

Statistical analysis was performed using the Statistical Package for Social Sciences (version 21.0; SPSS Inc, Chicago IL) at a statistical significance level of *P* < 0.05. Data were presented as frequency (%) for categorical variables and mean ± standard deviation (SD) for continuous variables. The differences in discrete and continuous variables across different tertiles of DED were compared using Chi-square test and one-way analysis of variance (ANOVA), respectively. Analysis of covariance (ANCOVA) was used to compare biochemical variables after adjustment for such confounders as age, gender, BMI, PA and energy intake. The multivariate multinomial logistic regression was performed with metabolic parameters, blood pressure as the dependent variable, and dietary energy density as the independent variable, and the ORs and 95% confidence intervals were obtained. The risk was reported in the three different models (Model I: crude, Model II: adjusted for age and sex, Model III: adjusted for age, BMI, sex, physical activity, SES and energy intake).

## Results

According to the results of this study, lower age and being single were accompanied with higher energy density; no difference in other demographic variables was observed (Table 1). Tables 2 and 3 compare the dietary energy, macronutrients, and intake of food groups by different tertiles of DED. Also, we observed the intake of higher energy, fat, saturated fatty acids, and mono- and poly-unsaturated fatty acids in those with higher tertiles of DED.

**Table 1:** General demographic characteristics of study participants by tertiles of DED

Variable	**All participants** ^**(*N*=335)**^		**Tertiles of dietary energy density**						
			**1**^**st**^ ^**0.32–0.83 kcal/g**^ ^**(*N*=112)**^		**2**^**nd**^ ^**0.83–1.02 kcal/g**^ ^**(*N*=111)**^		**3**^**rd**^ ^**1.02–1.93 kcal/g**^ ^**(*N*=112)**^		*P* value^*^
	**Mean**	**SD**	**Mean**	**SD**	**Mean**	**SD**	**Mean**	**SD**	
Age (y)	40.78	9.23	42.40	9.06	39.81	8.59	39.14	9.19	**0.017**
Education (≤ 12 y)	26	23.5	27	23.8	25	22.2	26	23.3	0.490
Marital status (% Single)	15	12.62	11	9.8	14	12.6	20	17.9	0.037^**^
Gender (% Male)	63.9	59.39	63	56.3	71	64	58	51.8	0.500^**^
BMI (kg/m^2^)	32.67	4.80	32.45	4.44	32.37	5.07	33.09	4.93	0.480
WC (cm)	106.78	9.62	106.62	9.63	106.98	9.22	106.34	10.04	0.880
FM (%)	33.81	9.13	32.06	8.41	33.97	10.11	34.90	8.78	0.190
FFM (%)	62.25	12.35	63.61	12.48	63.76	12.72	60.15	11.88	0.160
WHR	0.93	0.07	0.93	0.07	0.94	0.07	0.92	0.07	0.204
SES	9.96	2.51	10.35	2.48	9.90	2.53	9.75	2.45	0.370
Appetite	33.58	8.93	34.44	8.69	33.85	9.32	32.70	8.94	0.521
BMR (Kcal)	1904.99	396.79	1922.56	349.86	1938.13	358.83	1869.14	458.05	0.583
MetS status (%)	40.4	39.2	56.30	35.2	60.00	39.3	65.80	37.66	0.147^***^
SBP (mmHg)	122.99	16.35	122.68	14.88	125.87	14.80	118.98	10.63	0.004^***^
DBP (mmHg)	81.18	11.69	82.84	17.59	82.84	10.63	78.58	12.36	0.004^***^
FBS (mg/dl)	92.66	19.18	93.86	26.24	91.85	14.15	92.34	15.65	0.724^***^
TC (mg/dl)	191.45	36.64	190.49	37.24	193.88	40.89	190.67	32.26	0.745^***^
TG (mg/dl)	152,055	94.13	140.17	67.87	176.46	125.65	135.89	71.37	0.002^***^
HDL (mg/dl)	43.32	9.52	44.85	9.12	43.22	10.20	43.65	9.36	0.882^***^
LDL (mg/dl)	123.68	31.83	123.48	33.34	124.81	33.90	122.14	29.08	0.820^***^
Insulin (mIU/l)	16.17	13.66	14.73	10.32	14.85	9.19	18.06	18.07	0.181^***^
HOMA-IR	3.76	3.26	3.59	3.15	3.38	2.32	4.13	3.88	0.313^***^
QUICKI	0.32	0.03	0.33	0.04	0.32	0.02	0.32	0.00	0.270^***^

all data are mean (± SD) except marital status and gender, that is presented as the number and percent of single and males respectively in each group

*BMI* Body mass index, *WC* Waist Circumference, *FM* Fat Mass, *FFM* Fat Free Mass, *WHR* waist-to-hip ratio, *BMR* Basal Metabolic Rate, *PA* Physical Activity, *SES* Socio-economic status, TBW total body water, *TEFQ* Three eating factor questionnaire, *SBP* Systolic Blood Pressure, *DBP* Diastolic Blood Pressure, *TC* Total Cholesterol, *TG* Triglyceride, *HDL-C* High Density Lipoprotein Cholesterol, *LDL-C* Low Density Lipoprotein Cholesterol, *HOMA-IR* Homeostatic Model Assessment for Insulin Resistance, *QUICKI* Quantitative Insulin sensitivity Check Index; *DED* Dietary energy density

^*^ *P* values derived from One-Way ANOVA with Tukey’s post-hoc comparison

^**^ *P* values derived from chi-squared test

^***^ *P* values derived from One-Way ANOVA with Tukey’s post-hoc comparisons after adjustment for confounders (age, gender, BMI, PA and kcal); Bold values representstatistically significant threshold

**Table 2:** Dietary intakes of energy, macro and several micronutrients of study participants by tertiles of DED

Variable	**Tertiles of dietary energy density**						***P* value**
	**1**^**st**^ ^**0.32–0.83 kcal/g**^ ^**(*N*=112)**^		**2**^**nd**^ ^**0.83–1.02 kcal/g**^ ^**(*N*=111)**^		**3**^**rd**^ ^**1.02–1.93 kcal/g**^ ^**(*N*=112)**^		
	**Mean**	**SD**	**Mean**	**SD**	**Mean**	**SD**	
Energy (kcal/d)	2741.93	944.92	3005.91	962.61	3299.21	2291.09	**0.001**
CHO (%)	59.41	6.16	58.52	6.73	56.49	7.35	**0.042**
Protein (%)	13.33	2.11	13.24	1.79	12.63	1.93	0.079
Fat (%)	30.31	6.04	30.86	6.50	33.20	7.57	**0.035**
Fiber (g/d)	66.36	46.79	70.18	34.81	77.87	47.61	0.308
Cholesterol (mg/d)	275.48	151.93	305.11	253.03	313.31	181.13	0.330
SFA (g/d)	25.57	11.91	30.28	16.64	32.16	15.52	**0.003**
MUFA (g/d)	27.64	12.32	32.92	16.26	38.96	18.75	**< 0.001**
PUFA (g/d)	18.32	9.69	21.30	11.24	27.95	15.73	**< 0.001**

*CHO* Carbohydrate, *SFA* Saturated fatty acids, *MUFA* Mono-unsaturated fatty acids, *PUFA* Polyunsaturated fatty acids, *DED *Dietary energy density. Bold values represent statistically significant threshold

**Table 3:** Food groups intake of study participants by tertiles of DED

Variable	**Tertiles of dietary energy density**										
	**1**^**st**^ ^**0.32–0.83 kcal/g**^			**2**^**nd**^ ^**0.83–1.02 kcal/g**^			**3**^**rd**^ ^**1.02–1.93 kcal/g**^			*P* value^*^	*P* value^**^
	**N**	**Mean**	**SD**	**N**	**Mean**	**SD**	**N**	**Mean**	**SD**		
Fruits (g/d)	112	633.15	549.12	111	747.25	626.43	112	654.55	555.81	0.894	0.193
Vegetables (g/d)	112	306.95	174.92	111	335.43	218.55	112	366.11	329.79	0.377	0.139
Red processed meat (g/d)	112	30.21	26.68	111	30.10	30.18	112	31.72	34.53	0.493	0.714
MFP (g/d)	112	37.92	31.37	111	44.90	44.26	112	35.47	26.78	0.848	**0.022**
Dairy (g/d)	112	382.52	316.39	111	387.17	249.00	112	383.33	267.51	0.533	0.718
Nuts (g/d)	112	13.19	14.24	111	21.05	42.17	112	20.52	57.78	0.172	0.290
Legumes (g/d)	112	65.31	57.27	111	69.11	70.16	112	65.27	73.19	0.433	0.073
Grains (g/d)	112	541.06	253.28	111	560.82	257.28	112	537.56	210.01	0.369	0.681
carbonated drinks (g/d)	112	26.40	40.30	111	53.94	79.93	112	45.92	78.24	**0.009**	0.482
fruit juice (mg/d)	112	10.55	28.73	111	12.42	29.01	112	9.79	29.36	0.786	**0.002**
Milk (g/d)	112	32.42	65.39	111	39.16	73.55	112	30.89	57.14	0.607	0.715
Tea (ml/d)	112	1504.78	1174.70	111	794.95	559.75	112	534.39	481.15	**0.000**	**0.000**
Coffee (mg/d)	112	18.64	36.90	111	27.82	68.16	112	18.58	41.94	0.297	0.398

All data are mean (± SD)

*MFP* Meat, fish and poultry

^*^ *P* values derived from unadjusted ANCOVA

^**^ *P* values derived from ANCOVA after adjustment for confounders (age, gender, BMI, PA and energy intake). DED Dietary energy density. Bold values represent statistically significant threshold

**Fig. 1:**
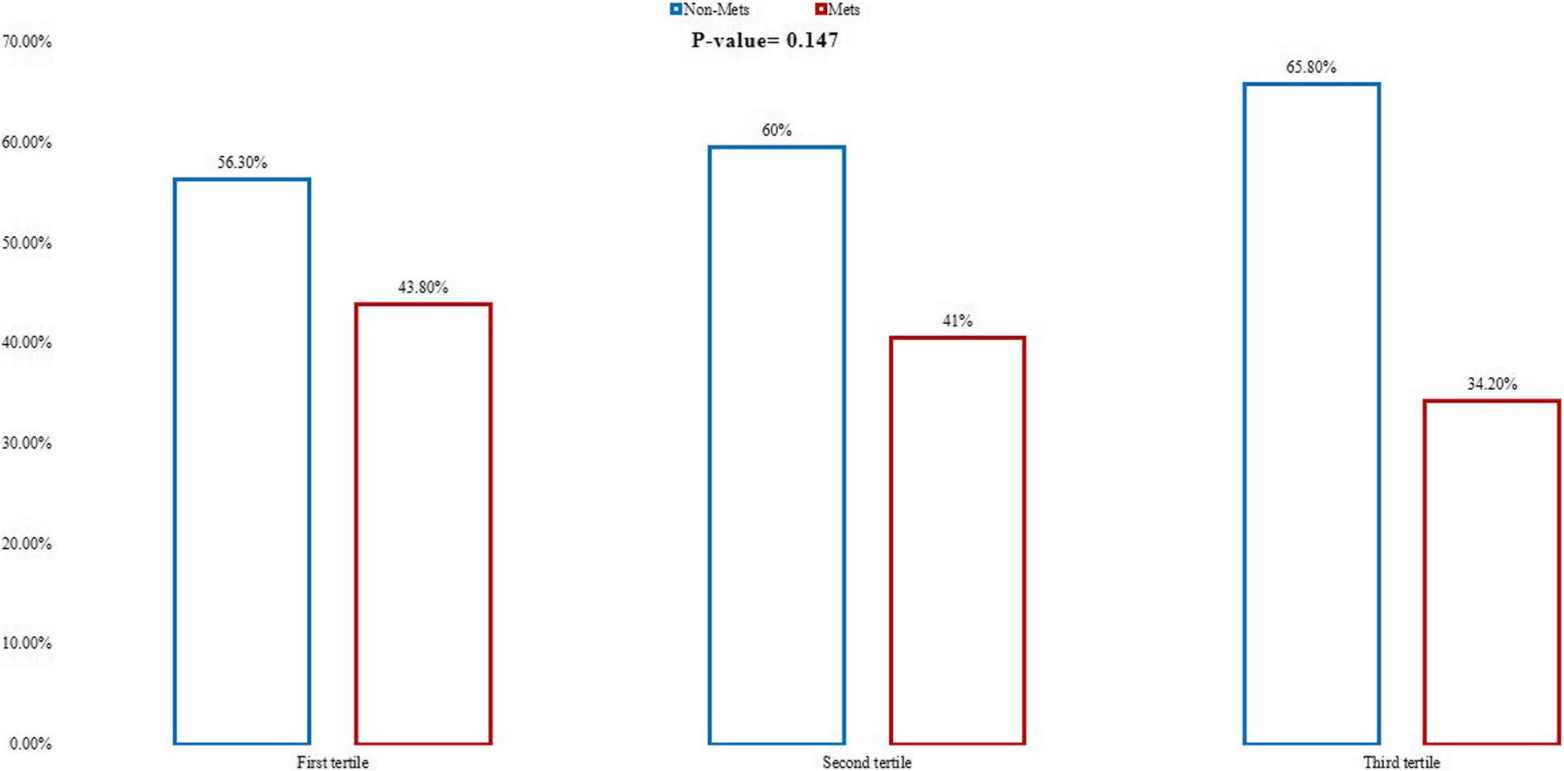
The prevalence of metabolic syndrome in different dietary energy density (DED) tertiles

Biochemical variables of participants by DED tertiles are presented in Table 4. Among the food groups, we witnessed a higher intake of meat, fish, poultry in the highest tertiles of DED (*P* < 0.05). No significant relationship was observed between DED I and DED II with SBP, FBS, LDL, insulin, TG, and HOMA-IR after controlling the confounders in the three models (*P* > 0.05). Also, people at the second tertile of DED I had lower DBP (OR = 0.921, *P* = 0.004). However, in the second tertile of DED I, QUICKI in model 2 had a significant increasing effect (OR = 1.640, *P* = 0.016). At the last tertile of DED I, the differences were not significant (*P* > 0.05). After adjusting for age and sex in the second tertile of DED II, TC and HDL levels were significantly different across the tertiles (*P* < 0.05), so that while TC levels significantly decreased (OR = 0.920, *P* = 0.024), HDL levels significantly increased (OR = 1.096, *P* = 0**.**033**)**. Moreover, during DED II tertiles, TC in the crude model had a significant decreasing effect (OR = 0.949, *P* = 0.036). QUICKI showed significant increase in second tertile of DED II both in models 2 and 3 (OR = 1.532 and OR = 1.771, respectively; *P* < 0.05). These results were not significant in the third tertile of DED II even after adjustment for covariates. As illustrated in the Fig. 1, chi-square test showed that there is no significant difference in the prevalence of metabolic syndrome among tertiles of DED (*p* = 0.147), but we witnessed a lower prevalence of MetS in the highest tertile of DED (34.2%) compared to the lowest tertile (43.8%).

**Table 4:** Biochemical variables of study participants by tertiels of DED

Variable		Tertiles of dietary energy density									
		**DED I**					**DED II**				
		**1**^**st**^ ^**0.32–0.83 kcal/g**^ ^**(*N*=112)**^	**2**^**nd**^ ^**0.83–1.02 kcal/g**^ ^**(*N*=111)**^		**3**^**rd**^ ^**1.02–1.93 kcal/g**^ ^**(N=112)**^		**1**^**st**^ ^**0.29–1.006 kcal/g**^ ^**(*N*=109)**^	**2**^**nd**^ ^**1.006–1.523 kcal/g**^ ^**(*N*=108)**^		**3**^**rd**^ ^**1.52–5.36 kcal/g**^ ^**(*N*=109)**^	
			OR(CI)	*P*-value	OR(CI)	*P*-value		OR(CI)	*P*-value	OR(CI)	*P*-value
SBP (mmHg)	Model I	**1 REF**	1.033 (1.00–1.06)	0.049	1.009 (0.97–1.04)	0.574	**1 REF**	0.989 (0.95–1.02)	0.484	1.005 (0.97–1.03)	0.733
	Model II		1.045 (1.00–1.09)	**0.038**	1.008 (0.96–1.04)	0.690		1.008 (0.96–1.04)	0.679	1.020 (0.98–1.06)	0.302
	Model III		1.069 (1.02–1.12)	**0.005**	1.021 (0.97–1.06)	0.352		0.996 (0.95–1.04)	0.872	0.977 (0.92–1.02)	0.354
DBP (mmHg)	Model I	**1 REF**	0.955 (0.91–0.99)	**0.045**	0.960 (0.92–1.00)	0.058	**1 REF**	1.001 (0.95–1.04)	0.966	1.001 (0.96–1.04)	0.958
	Model II		0.967 (0.91–1.02)	0.255	0.965 (0.91–1.01)	0.171		0.999 (0.94–1.05)	0.961	0.984 (0.934–1.03)	0.545
	Model III		0.921 (0.87–0.97)	**0.004**	0.954 (0.90–1.00)	0.084		1.010 (0.95–1.06)	0.713	1.030 (0.96–1.09)	0.343
FBS (mg/dl)	Model I	**1 REF**	0.995 (0.95–1.03)	0.787	1.007 (0.97–1.03)	0.667	**1 REF**	1.005 (0.97–1.04)	0.797	1.01 (0.98–1.04)	0.435
	Model II		0.985 (0.93–1.03)	0.564	1.017 (0.98–1.05)	0.786		1.01 (0.96–1.06)	0.576	1.003 (0.96–1.04)	0.891
	Model III		0.991 (0.94–1.04)	0.709	1.005 (0.96–1.05)	0.815		0.985 (0.92–1.04)	0.608	0.998 (0.93–1.06)	0.960
TC (mg/dl)	Model I	**1 REF**	0.982 (0.94–1.01)	0.327	0.993 (0.95–1.03)	0.705	**1 REF**	0.949 (0.90–0.99)	**0.036**	0.971 (0.92–1.01)	0.200
	Model II		0.959 (0.89–1.02)	0.203	0.999 (0.952–1.04)	0.983		0.920 (0.85–0.98)	**0.024**	0.973 (0.92–1.02)	0.290
	Model III		1.005 (0.99–1.01)	0.474	1.005 (0.99–1.01)	0.416		1.009 (0.99–1.02)	0.223	1.004 (0.98–1.02)	0.620
TG (mg/dl)	Model I	**1 REF**	1.004 (0.99–1.01)	0.231	1.000 (0.99_1.00)	0.956	**1 REF**	1.01 (1.00–1.01)	0.034	1.005 (0.99–1.01)	0.280
	Model II		1.009 (0.99–1.02)	0.131	0.998 (0.98–1.00)	0.760		1.010 (1.00–1.03)	**0.023**	1.004 (0.99–1.01)	0.495
	Model III		0.999 (0.99–1.00)	0.859	0.998 (0.99–1.00)	0.580		1.003 (0.99–1.01)	0.534	1.005 (0.99–1.01)	0.325
HDL (mg/dl)	Model I	**1 REF**	1.026 (0.97–1.07)	0.313	1.003 (0.95–1.05)	0.901	**1 REF**	1.03 (0.97–1.09)	0.252	1.016 (0.96–1.07)	0.572
	Model II		1.071 (0.99–1.15)	0.083	0.998 (0.93–1.06)	0.954		1.096 (1.00–1.19)	**0.033**	1.031 (0.96–1.10)	0.377
	Model III		1.019 (0.96–1.07)	0.495	0.995 (0.94–1.04)	0.830		0.996 (0.94–1.05)	0.887	1.012 (0.95–1.07)	0.711
LDL (mg/dl)	Model I	**1 REF**	0.952 (0.74–1.22)	0.703	0.911 (0.71–1.15)	0.443	**1 REF**	0.989 (0.77–1.26)	0.929	0.880 (0.68–1.13)	0.323
	Model II		0.952 (0.74–1.22)	0.703	0.911 (0.71–1.15)	0.443		0.997 (0.77–1.28)	0.983	0.885 (0.68–1.14)	0.352
	Model III		0.692 (0.38–1.23)	0.212	1.08 (0.62–1.84)	0.775		0.556 (0.27–1.14)	0.110	0.629 (0.29–1.33)	0.228
Insulin (mIU/l)	Model I	**1 REF**	1.031 (0.82–1.28)	0.786	1.092 (0.94–1.26)	0.244	**1 REF**	1.04 (0.89–1.22)	0.581	1.058 (0.89–1.04)	0.507
	Model II		0.920 (0.70–1.19)	0.535	1.160 (0.96–1.39)	0.120		1.15 (0.93–1.41)	0.181	1.086 (0.89–1.32)	0.407
	Model III		0.974 (0.72–1.30)	0.856	1.057 (0.83–1.34)	0.649		0.943 (0.66–1.34)	0.747	1.021 (0.69–1.49)	0.913
HOMA-IR	Model I	**1 REF**	0.688 (0.29–1.60)	0.388	0.707 (0.38–1.30)	0.269	**1 REF**	0.809 (0.40–1.60)	0.543	0.784 (0.38–1.60)	0.505
	Model II		0.897 (0.34–2.32)	0.823	0.546 (0.24–1.21)	0.139		0.503 (0.20–1.26)	0.145	0.664 (0.28–1.53)	0.338
	Model III		0.713 (0.25–1.98)	0.518	0.766 (0.34–1.72)	0.520		0.954 (0.26–1.4)	0.943	0.764 (0.18–2.08)	0.706
QUICKI	Model I	**1 REF**	1.87 (1.82–1.92)	0.057	0.005 (0.00–5.7)	0.438	**1 REF**	1.91 (1.61–2.20)	0.187	1.890 (1.45–2.60)	0.822
	Model II		1.64 (1.41–1.83)	**0.016**	0.008 (0.00–0.02)	0.353		1.532 (1.11–1.67)	**0.018**	0.002 (0.00–0.004)	0.493
	Model III		1.46 (1.33–1.63)	0.062	0.004 (0.00–0.00)	0.524		1.771 (1.23–2.05)	**0.030**	1.02 (0.90–1.01)	0.139

The multivariate multinomial logistic regression was used for estimation of ORs and confidence interval (CI).Model I: crude, Model II: adjusted for age and sex, Model III: adjusted for age, BMI, sex, physical activity, SES and energy intake

*DED I* Energy density from foods and beverages, *DED II* Energy density from just foods, *SBP* Systolic Blood Pressure, *DBP* Diastolic Blood Pressure, *TC* Total Cholesterol, *TG* Triglyceride, *HDL-C* High Density Lipoprotein Cholesterol, *LDL-C* Low Density Lipoprotein Cholesterol, *HOMA-IR* Homeostatic Model Assessment for Insulin Resistance, *QUICKI*, Quantitative Insulin sensitivity Check Index, *OR* Odds ratio, *CI* Confidence interval, DED Dietary energy density. Bold values represent statistically significant threshold

## Discussion

To the best of our knowledge, for the first time in East Azerbaijan province, this study evaluated the association between DED and odds of MetS and cardiovascular disease among individuals with obesity in Tabriz and Tehran, Iran. Moreover, Food and beverage items with a high water content can significantly lower the DED of foods, meals, and the entire diet because water provides a lot of weight without providing any energy [48], given the mean daily fluid intake specially water of subjects in Tabriz, the area where the study was conducted, was lower than recommended values [49]. Therefore the current study calculated DED with and without beverages. We revealed that a high DED was associated with lower DBP and TC levels but higher HDL levels and QUICKI after controlling for the confounders. Also, a high DED was associated with lower prevalence of MetS among adults with obesity but it was not statistically significant. In the study by Shahinfar et al*.* [30], being at higher tertile of DED was associated with lower SBP and DBP among general adult population. Also, Takeda et al*.* [22] reported a non-significant reduction in SBP in higher DED quintiles of patients with diabetes. This might be due to higher intake of nuts in higher tertiles of DED. Several previous studies have revealed the positive effects of nuts intake in lowering blood pressure. In a population-based study by Yazdekhasti et al*.* [50] among 9,660 Iranian adults, high dietary intake of nuts was associated with lower blood pressure and lower risk of hypertension. In a meta-analysis of 21 randomized controlled trials, high total nuts consumption was associated with lower blood pressure [51]. Although the difference in consumption of nuts was not statistically significant in our study, the difference was nutritionally meaningful. Jenkins et al. propose a dose response in which ~ 7 g of almonds per day lowers LDL cholesterol by approximately 1%, resulting in a 2% risk reduction for CHD [52], in the current study the difference of nut consumption in first tertile and third tertile of DED is almost 7 gr so it can have positive effects on blood pressure. Lower TC levels in the highest DED tertile can also be attributed to higher intakes of monounsaturated fatty acid (MUFA), polyunsaturated fatty acid (PUFA), or fiber in our study. Although the difference in fiber intake was not statistically significant, the difference was meaningful from a nutritional point of view. High intake of MUFA and PUFA in higher tertiles of DED was also reported in the study by Bezshahi et al*.* among Iranian adults [46]. The positive effects of PUFA in reducing triglyceride is due to increased hepatic carnitine palmitoyl transferase and reduced hepatic phosphatidate phosphohydrolase activities [53]. It is also suggested that PUFA exert their beneficial effects by up-regulation of the transcription factor peroxisome proliferator-activated receptor α gene expression and down-regulation of lipogenic gene expressions. Also, PUFA suppress the nuclear abundance and expression of sterol regulatory element binding protein-1 and reduce the DNA-binding activities of nuclear factor Y, Sp1, and possibly hepatic nuclear factor-4 [54]. However, the results of different studies in this issue are inconsistent; for example in the study by Azadbakht et al*.* [55], higher intake of energy dense foods was associated with higher levels of serum HDL and TG in female nurses. Meanwhile, in another study, no significant association was reported between DED and serum HDL and TG levels in free-living Japanese women [56].

In our study, we did not observe any difference in obesity measurements, including BMI, WC, or WHR in different DED tertiles. Several previous studies also reported no significant difference between BMI and WC according to DED categorization. For example, Bazshahi et al*.* [46] did not report any association between DED and anthropometric variables or body composition among healthy general population. Similar results were also found in the study by Maddahi et al*.* among the women with overweight or obesity [29], in the study by Sasaki et al*.* [18] in general Japanese population, and in the study by Shahinfar et al*.* among Tehranian older adults [30]. It seems that DED may not be associated with BMI, but rather it is associated with odds of obesity. In a recent systematic review and meta-analysis of observational studies, DED was related to increased adiposity risk, greater body weight change, but not BMI and WC [25]. However, several previous studies reported a positive association between DED and WC [31, 57].

**Fig. 2:**
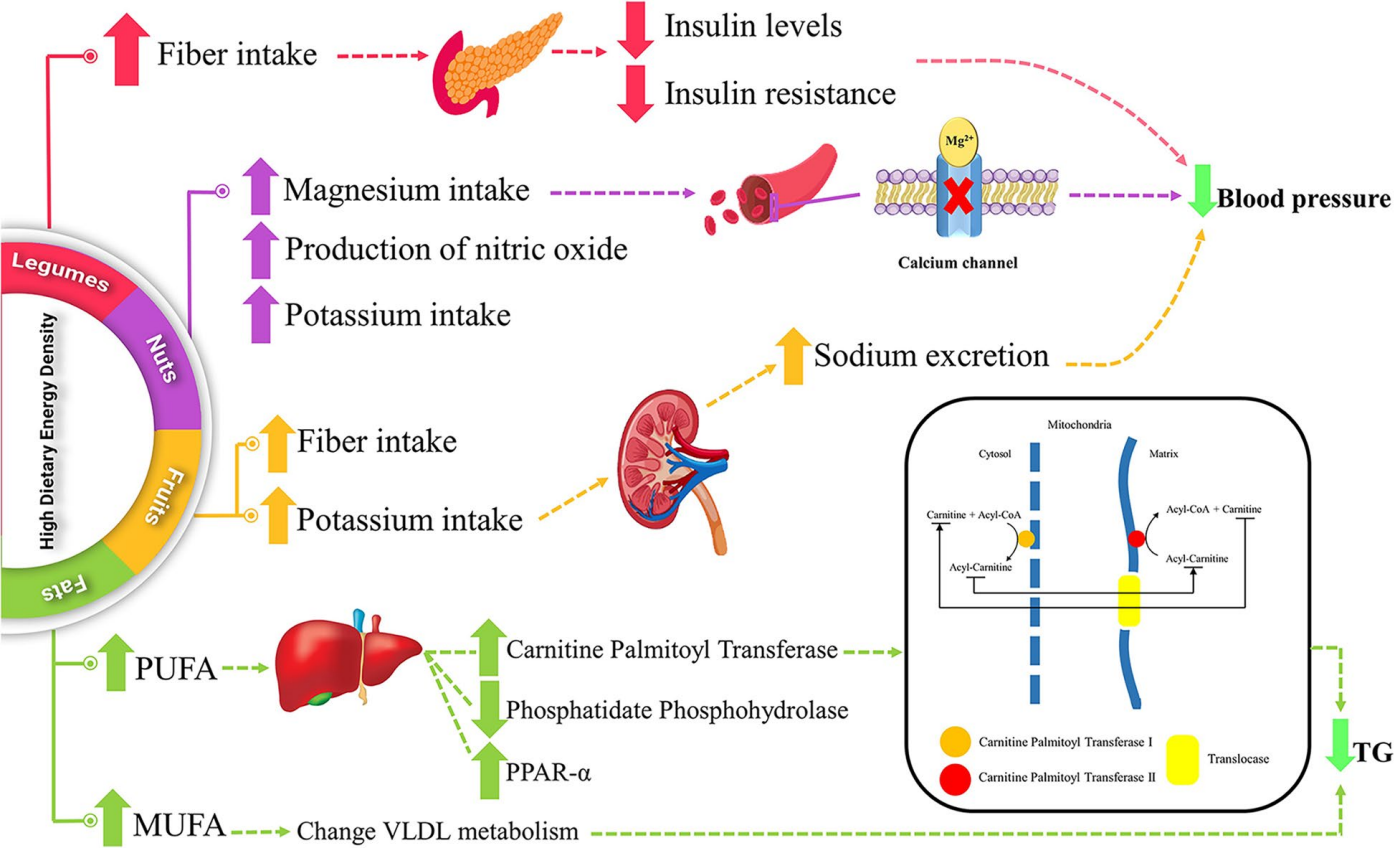
Mechanistic pathways of the possible effects of dietary energy density (DED) in metabolic parameters and blood pressure

As mentioned above, there is an inconsistency in the results of different studies regarding the association between DED and metabolic or anthropometric risk factors of obesity or MetS. This might be attributed to such factors as gender, age, and eating habits of the people. Gender can affect the body composition of people and cause different results. In the present study, apparently healthy adults with obesity were examined; so, it is not expected to see significant changes in all the biochemical levels of the variables. These conflicting results might be due to different dietary assessment tools and difference in the demographic characteristics of the studied populations. For example, some of the studies, including our study, used FFQ for dietary assessment [29, 30], but some others used 24-h recall method [31, 58, 59], which is not a reflection of long-term habitual dietary intakes. Moreover, one important issue is the inclusion or exclusion of beverages in DED calculation. While several studies excluded beverages because they believed that inclusion of beverages might weaken the association of DED with health outcomes [18, 22, 30], some others included beverages because they believed that beverages have an important role in one’s usual energy intake [58, 60, 61]. Even after excluding the beverage intake from DED calculation, the energy intakes from beverages is an important confounder that its effect should be controlled as performed by several studies [56, 62]. Some of the mechanistic pathways of the role of DED in modifying metabolic parameters are presented in Fig. 2.

This study had several limitations. First, the cross-sectional design of the study makes the causal inference difficult; longitudinal studies are needed to better elucidate the cause-effect associations. Second, the absence of a control group impeded the control of confounding factors. Third, the FFQ was not initially designed to evaluate DED. Forth, recall bias is possible due to the subjective character of questionnaire-based data including FFQ and also VAS that can affected by fasting state considering the fact that the orexigenic hormone, ghrelin, has been proposed as a modifiable appetite hormone via altered feeding patterns. After a fasting intervention, ghrelin may peak later in the morning or be reduced at the start of the day [63], As a result, Fasting state in the morning will probably not affect the hormonal responses of appetite and response to VAS [63, 64].

The present study also had several strengths. This is the first relatively large-scale study examining the association between MetS and DED among Iranian population with obesity. Meanwhile, the multivariate multinomial logistic regression was adjusted by a considerable number of potential confounding factors in three models; this improved the reliability of the results.

In conclusion, we witnessed lower SBP, DBP, and TG levels in higher tertiles of DED among 335 individuals with obesity. These inverse associations might be due to higher intakes of PUFA, MUFA, and MFP factor in higher categories of DED. However, further well-designed studies are warranted to elucidate better results.

## Data Availability

The datasets generated and/or analyzed during the current study are not publicly available due privacy and ethical considerations but are available from the corresponding author on reasonable request.
